# Hemiepiphysiodesis with tension band plate in idiopathic genu valgum: Insights on indications, outcomes, and follow-up from a systematic review and meta-analysis of more than 600 cases

**DOI:** 10.1051/sicotj/2026047

**Published:** 2026-10-08

**Authors:** Luca Bianco Prevot, Francesco Natoli, Domenico Curci, Eleonora Caboni, Laura Mangiavini, Fabio Verdoni

**Affiliations:** 1 IRCCS Ospedale Galeazzi – S. Ambrogio Via Cristina Belgioioso 173 20157 Milan Italy; 2 Residency Program in Orthopedics and Traumatology, University of Milan Via Festa del Perdono 7 20122 Milan Italy; 3 Laboratory of Applied Biomechanics Department of Biomedical Sciences for Health, University of Milan Milan Italy

**Keywords:** Genu valgum, Hemiepiphysiodesis, Guided growth, Tension band plate

## Abstract

*Aims*: Hemiepiphysiodesis with tension band plates is widely used for the correction of idiopathic genu valgum in skeletally immature patients. Although widely adopted, considerable heterogeneity persists regarding indications, timing of intervention, and radiographic outcome assessment, limiting the development of standardized clinical pathways. *Methods*: A systematic review and meta-analysis were conducted in accordance with PRISMA guidelines. Studies reporting radiographic and clinical outcomes of hemiepiphysiodesis with tension band plates in idiopathic genu valgum were included. The study evaluates mechanical axis deviation (MAD), mechanical lateral distal femoral angle (mLDFA), and medial proximal tibial angle (MPTA), indication for surgery, timing of implant removal, complications, and follow-up interval. The risk of bias and quality of evidence were assessed using Cochrane guidelines. *Results*: A total of 11 studies, including more than 600 treated knees, were analyzed. Hemiepiphysiodesis resulted in significant improvement in coronal plane alignment. MAD improved by a pooled mean difference of 12.4 mm (*p* = 0.002). mLDFA shoed a mean correction of 5.2**°** (*p* = 0.004), while MPTA improved by 3.6° (*p* = 0.01). The overall complication rate was low (6.8%), mainly related to transient implant-site irritation and mild rebound growth, with no permanent physeal damage reported. *Conclusions*: Hemiepiphysiodesis with tension band plates provides safe, predictable, and clinically meaningful correction of idiopathic genu valgum in skeletally immature patients, supporting its role as a first-line surgical option and highlighting the importance of standardized radiographic monitoring and follow-up strategies to optimize timing of implant removal and minimize the risk of over- or under-correction.

## Introduction

Angular deformities of the lower limbs are a common condition in the pediatric population and may represent either a physiological stage of growth or the manifestation of an underlying skeletal disorder. Among these, genu valgum is one of the most frequent [1]. While a mild degree of valgus alignment is considered part of normal development, excessive or persistent deviation can lead to gait abnormalities, pain, patellofemoral dysfunction, and an increased risk of early joint degeneration [2].

In patients with remaining growth potential, guided growth correction has progressively replaced osteotomy as the first-line treatment for coronal plane deformities, owing to its minimal invasiveness, reversibility, and safety. The biomechanical principle underlying temporary hemiepiphysiodesis consists of applying an asymmetric constraint to one side of the physis, slowing its growth while allowing the opposite side to continue, thus producing a gradual correction of the mechanical axis [3]. In recent years, the tension band plate system has become the most widely used technique due to its effectiveness and lower risk of physeal injury. Nonetheless, considerable variability remains in surgical technique and postoperative management across centers, and alternative implants such as percutaneous transphyseal screws or traditional staples continue to be used in some institutions [4]. Such variability reflects the absence of consensus regarding the most effective and safest approach, particularly in younger children and in non-idiopathic cases. Moreover, most of the available literature consists of small, retrospective studies with limited follow-up and heterogeneous inclusion criteria. Evidence regarding correction rates, optimal timing for implantation and removal, the incidence of rebound deformity, and complication rates remains variable and sometimes conflicting [5, 6]. A comprehensive synthesis capable of guiding surgical practice in a standardized manner is still lacking.

In light of these considerations, the present systematic review aims to evaluate the complications and radiographic outcomes of guided growth using the tension band plate technique in pediatric idiopathic genu valgum, to clarify the surgical indications, and to define the most appropriate follow-up timing for monitoring patients during growth.

## Methods

The literature search was performed on 06/09/2025 across three major databases – PubMed (501 results), Embase (690 results), and Web of Science (83 results) – without applying time or language filters. The following search strategy was used: (guided growth OR hemiepiphysiodesis OR temporary hemiepiphysiodesis OR tension band plate OR eight-plate OR 8-plate OR eight plate) AND (genu valgum OR genu varum OR knee deformity OR knee deformities OR coronal plane OR frontal plane). The study protocol was prospectively registered on PROSPERO (ID: CRD420251182241). The review followed the methodological recommendations outlined in the PRISMA statement and Cochrane Handbook for Systematic Reviews [7]. Two independent reviewers (LB.P. and F.N.) screened all records and extracted data, with any disagreements resolved by discussion and consultation with a third reviewer (F.V.).

In the first screening phase, titles and abstracts were assessed according to predefined inclusion criteria: clinical studies (minimum of ten knees), published in English, evaluating the results of the tension bend plate for the correction of genu valgus. Exclusion criteria included studies published in other languages, literature reviews, preclinical or in vitro investigations, case reports, case series with fewer than ten knees, conference abstracts, and studies lacking clinical outcome data.

Full-text articles that met these criteria were retrieved for detailed assessment. Data were extracted from the text, tables, and figures of the included studies and summarized for analysis. The extracted variables included: author, year of publication, study design, age, sex, body mass index (BMI), site of plate implantation, pre- and post operative lateral distal femoral angle (LDFA), pre- and postoperative medial proximal tibial angle (MPTA), time to correction, pre- and postoperative hip-knee-ankle (HKA) angle, degree of deformity correction, intermalleolar distance (IMD), indication for surgery, timing of implant removal, complications, and follow-up interval.

## Statistical analysis

A single-arm meta-analysis was conducted, including non-comparative studies reporting preoperative and postoperative radiographic parameters, specifically the LDFA and the MPTA. For each study, means and standard deviations (SD) of preoperative and postoperative values were extracted. When studies reported these values separately, the mean change (Δ) was calculated as the difference between postoperative and preoperative means. Pooled analyses were performed using a random-effects model according to the DerSimonian and Laird method, which accounts for both within-study and between-study variability [8]. Heterogeneity among studies was assessed using Cochran’s Q statistic, the between-study variance (*τ*^2^), and the *I*^2^ statistic, which quantifies the proportion of total variability attributable to heterogeneity rather than chance. Heterogeneity was interpreted as low (*I*^2^ < 25%), moderate (25–75%), or high (>75%) [9]. All analyses were performed using Python (version 3.11), through custom scripts implementing standard meta-analytic procedures. Results were graphically represented using forest plots.

## Results

The database search initially retrieved 1274 articles. After the removal of duplicates and the subsequent screening of titles, abstracts, and full texts, a total of 11 studies met the inclusion criteria and were incorporated into the final analysis (Figure 1) [6, 10–19].

**Figure 1:**
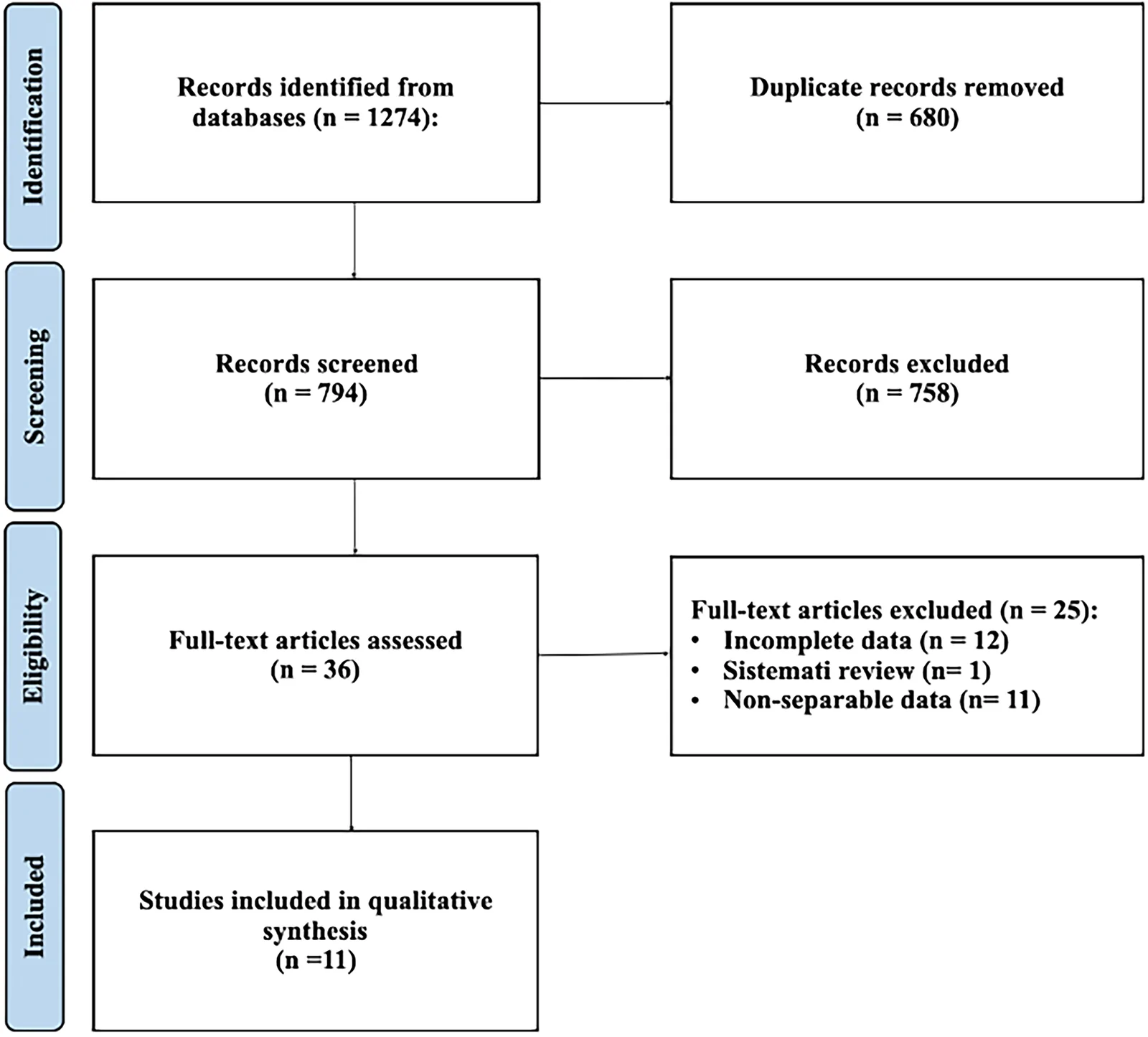
Prisma flow chart.

This work included 11 studies; 1 was a randomized controlled trial (RCT), and 10 were retrospective non-randomized studies. Overall, the included studies involved 363 patients who underwent tension band plate implantation for the treatment of genu valgum, accounting for a total of 678 treated knees. Of the included studies, 3 studies included patients treated exclusively with distal medial femoral (DMF) hemiepiphysiodesis; 3 studies included patients who underwent DMF or medial proximal tibial (MPT) hemiepiphysiodesis; 2 studies included patients treated with DMF or MPT or combined DMF and MPT hemiepiphysiodesis; and 3 studies included patients treated with DMF or MPT hemiepiphysiodesis. Among them, 66.0% underwent DMF hemiepiphysiodesis, 13.9% underwent MPT hemiepiphysiodesis, and 20.0% received combined DMF and MPT hemiepiphysiodesis. The mean correction time was 13.2, SD 5.3 months, and the mean age of the analyzed population was 11.7, SD 2.6. Table 1 reports the study data and the mean values of pre- and pos toperative LDFA, MPTA, and HKA angles.

**Table 1:** Pre- and post-operative radiographic parameters across the included studies.

Author	Study type	Knees	Age (SD)	LDFA pre (SD)	LDFA post (SD)	MPTA pre (SD)	MPTA post (SD)	HKA angle pre (SD)	HKA angle post (SD)
Ghaznavi et al. (2022) [10]	Retro	90	11.3 (1,3)	84.2 (2.5)	92.3 (2.9)	91.1 (1.9)	88.4 (2.9)	6.7 (2.7)	−4.3 (3.3)
Boero et al. (2011) [11]	Retro	60	12.5 (1.8)	n.r.	n.r.	n.r.	n.r.	14.0 (2.1)	5.0 (1.2)
Vaishya et al. (2017) [12]	Retro	35	10.0 (7,8)	n.r.	n.r.	n.r.	n.r.	22.0 (5.2)	86.1 (1.9)
Salvato et al. (2024) [13]	Retro	97	12.5 (1,9)	84.0 (3.4)	91.9 (3.8)	90.7 (2.7)	88.6 (2.6)	6.5 (3.0)	−1.8 (3.1)
Zajonz et al. (2017) [14]	Retro	186	12.0 (2.2)	78.0 (2.5)	83.7 (3.2)	90.8 (3.3)	88.3 (2.5)	12.0 (2.7)	2.3 (4.0)
Burghardt et al. (2010) [15]	Retro	13	9.5 (2.5)	85.6 (2.3)	89.5 (3.6)	92.5 (2.7)	88.0 (4.6)	n.r.	n.r.
Jelinek et al. (2011) [16]	Retro	33	11.6 (3.8)	84.0 (3.0)	89.0 (3.0)	93.0 (4.0)	88.0 (4.0)	n.r.	n.r.
Park et al. (2022) [17]	Retro	73	11.3 (2,2)	82.8 (2.4)	89.7 (2.8)	93.6 (2.9)	87.2 (2.3)	n.r.	n.r.
Gottliebsen et al. (2013) [18]	RCT	20	10.1 (2)	85.5 (1.3)	92.0 (1.3)	89.1 (1)	90.0 (2.5)	n.r.	n.r.
Park et al. (2018) [6]	Retro	60	11.7 (1.6)	83.4 (2.2)	89.3 (2.5)	90.9 (1.2)	86.5 (1.9)	n.r.	n.r.
Guzman et al. (2011) [19]	Retro	47	12.5 (1.5)	78.7 (3.4)	n.r.	n.r.	n.r.	11.2 (2.7)	6.0 (3.7)

Abbreviations: SD, standard deviation; LDFA, lateral distal femoral angle; MPTA, medial proximal tibial angle; HKA, hip-knee-ankle angle; Retro, retrospective study; RCT, randomized controlled trial; n.r., not reported. Values are expressed as means (SD) when appropriate.

### Surgical indications

The criteria for surgical indication were explicitly reported in only 5 of the 10 included studies. Considerable variability emerged among authors regarding the thresholds used to recommend intervention. Some studies based the indication on radiographic parameters, typically defining surgical candidacy for patients presenting with a valgus knee deformity exceeding 6° to 10°. Others relied primarily on clinical evaluation, recommending surgery when the intermalleolar distance (IMD) exceeded 10 cm, or greater than 7 cm in symptomatic or progressively worsening cases. In a few reports, additional functional symptoms such as anterior knee pain, patellofemoral instability, gait disturbance, or relevant cosmetic concerns were also considered valid indications. Only one study incorporated the assessment of residual growth potential, requiring at least 12 months of expected growth as evaluated through hand radiography.

### Follow-up

The follow-up protocol varied among the included studies. In 4 studies, patients underwent clinical and radiographic evaluations every 2 to 3 months, with some authors specifying radiographic follow-up every 2 months and others every 3 months or at the end of clinical correction. 1 study reported follow-up visits every 4 months, while in the remaining papers, the follow-up schedule was not clearly specified. Table 2 reports the mean correction rates, the criteria used for surgical indication, the complications, and the follow-up protocols.

**Table 2:** Summarizes the characteristics of the included studies, detailing study design, sample size, surgical indications, complications, and follow-up intervals.

Author	Study type	Knees	Correction rate (SD)	Indications for surgery	Complications	Control intervals
Ghaznavi et al. (2022) [10]	Retro	90	n.r.	Gait *impairment*, genu valgum deformity ≥6°, or lower but progressive valgus angles.	2 overcorrection, 1 screw loosening	every 2 months + X-Ray
Boero et al. (2011)[11]	Retro	60	n.r.	Deformity > than 10°	no complication	every 3 months
Vaishya et al. (2017) [12]	Retro	35	18.0 (6.0)	n.r.	1 superficial infection, 2 overcorrection	every 3 months + X-Ray
Salvato et al. (2024)[13]	Retro	97	17.9 (7.6)	n.r.	no complication	every 2 months + X-Ray at the end of correction
Zajonz et al. (2017) [14]	Retro	186	13.0 (6.4)	DIM >10 cm	3 superficial infection, 3 overcorrection	n.r.
Burghardt et al. (2010) [15]	Retro	13	13.2 (7.4)	n.r.	n.r.	n.r.
Jelinek et al. (2011) [16]	Retro	33	11.9 (6.8)	n.r.	1 knee ROM reduction, 4 overcorrection	n.r.
Park et al. (2022) [17]	Retro	73	9.0 (2.1)	n.r.	n.r.	n.r.
Gottliebsen et al. (2013)[18]	RCT	20	11.2 (0.9)	DIM >7 cm with sympthomps	n.r.	n.r.
Park et al. (2018)[6]	Retro	60	9.5 (2.8)	n.r.	n.r.	every 4 months
Guzman et al. (2011) [19]	Retro	47	13.5 (3.7)	Deformity > than 10°, with joint pain, patellofemoral instability, gait disturbance, or cosmetic concerns	3 recurvatum deformity	n.r.

Abbreviations: SD, standard deviation; Retro, retrospective study; RCT, randomized controlled trial; IMD, intermalleolar distance; ROM, range of motion; n.r., not reported. Values are expressed as means (SD), when appropriate.

### Meta-analysis Outcomes: LDFA and MPTA

A total of 572 knees from 8 studies were included in the quantitative synthesis. The mean preoperative LDFA was 83.4° (95% CI 80.9–86), increasing postoperatively to 89.7° (95% CI 87.1–92.2). The mean change (ΔLDFA) between pre- and postoperative measurements was +6.3° (95% CI 5.5–7.1) (Figure 2).

**Figure 2:**
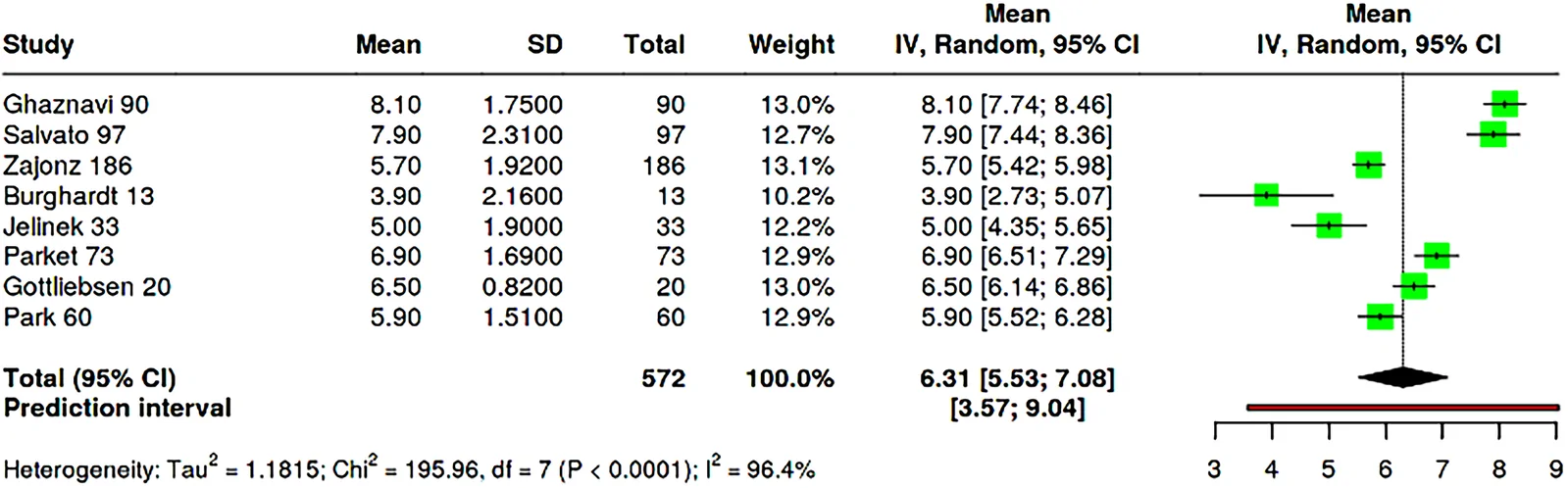
Forest plot of the analysis on ΔLDFA.

The preoperative MPTA showed a mean of 88.1° (95% CI 87.4–88.8), which increased postoperatively to 91.4° (95% CI 90.5–92.2). The overall mean change (ΔMPTA) was –3.3° (95% CI −4.6 to −2.1) (Figure 3).

**Figure 3:**
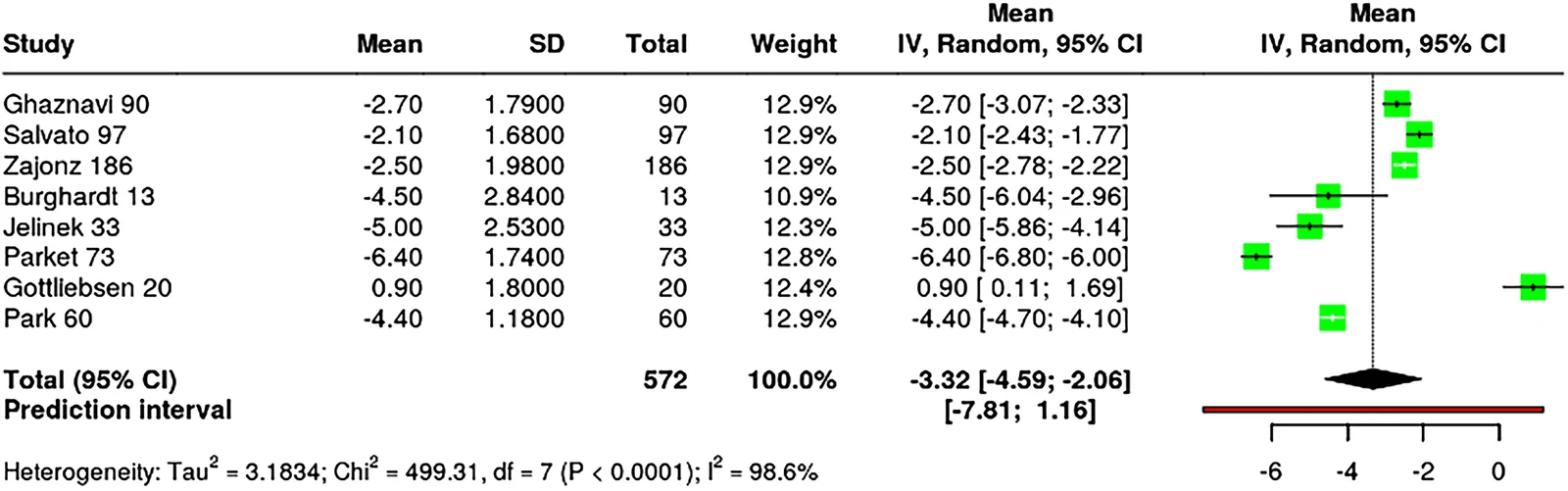
Forest plot of the analysis on ΔMPTA.

### Hip-knee-ankle angle

The HKA angle was reported in 6 studies, including a total of 515 treated knees. The pooled mean preoperative HKA angle was 12.1° SD 5.7, while the postoperative HKA angle decreased to 2.2° SD 3.6. The overall mean change between pre- and postoperative measurements was 9.8° SD 4.3 (Figure 4).

**Figure 4:**
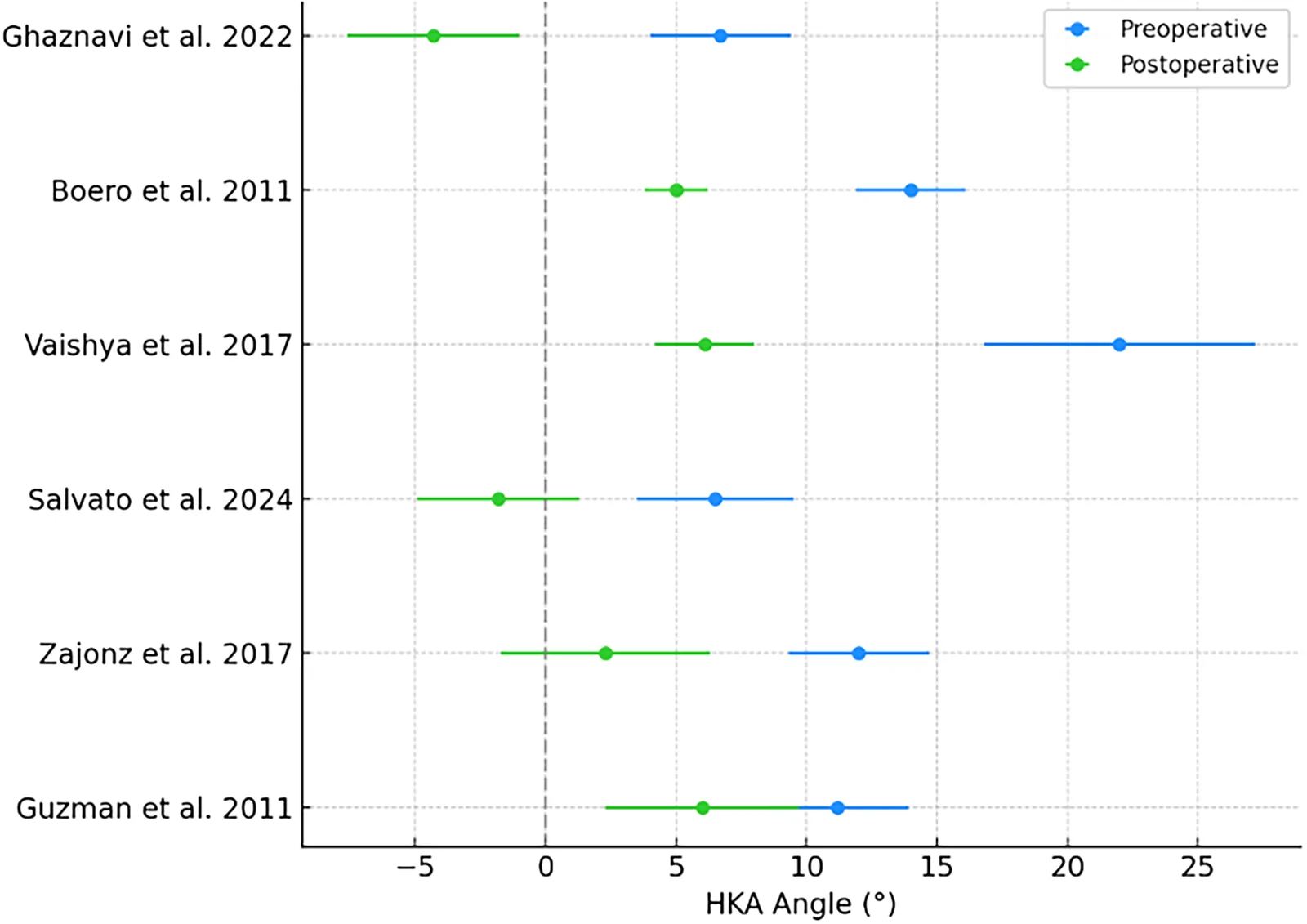
Forest plot illustrating the preoperative and postoperative HKA angles reported in the included studies. Each point represents the mean value with the corresponding standard deviation. Blue markers indicate preoperative HKA angles, while green markers represent postoperative values.

### Complications

Complications were described in 7 of the included papers, with a total of 20 adverse events recorded. The most frequent complication was overcorrection, reported in 5 studies, with a total of 11 cases. Superficial infections occurred in 4 patients, all successfully managed with conservative treatment. Other less frequent events included screw loosening (1 case), reduction in range of motion (1 case), and recurvatum deformity (3 cases). Notably, 4 studies explicitly stated that no complications were observed during follow-up. The overall complication rate was 3.6%.

### Risk of bias and quality of evidence

Reviewers evaluated the studies considered in the meta-analysis using the revised tool for Risk of Bias in randomized trials (RoB 2.0) for randomized studies and the Risk of Bias In Non-randomised Studies of Interventions (ROBINS) tool for non-randomized clinical studies, as recommended by Cochrane [20]. The evaluation using the RoB2 tool showed 1 study falling in the “Some concerns” category, while the evaluation using the ROBINS-I tool showed an overall heterogeneous quality of the studies, with 3 papers falling in the “Moderate” category and 2 papers falling in the “serious” category.

Detailed results are shown in Figures 5 and 6.

**Figure 5:**
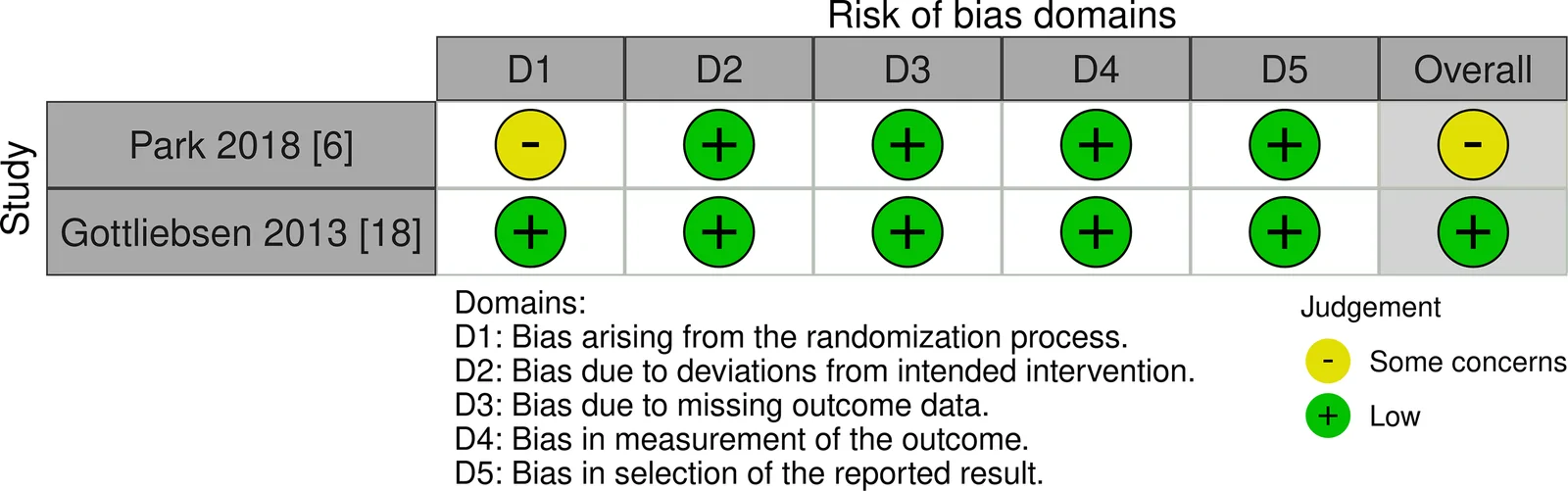
Risk of bias assessments according to RoB 2.0 tools.

**Figure 6:**
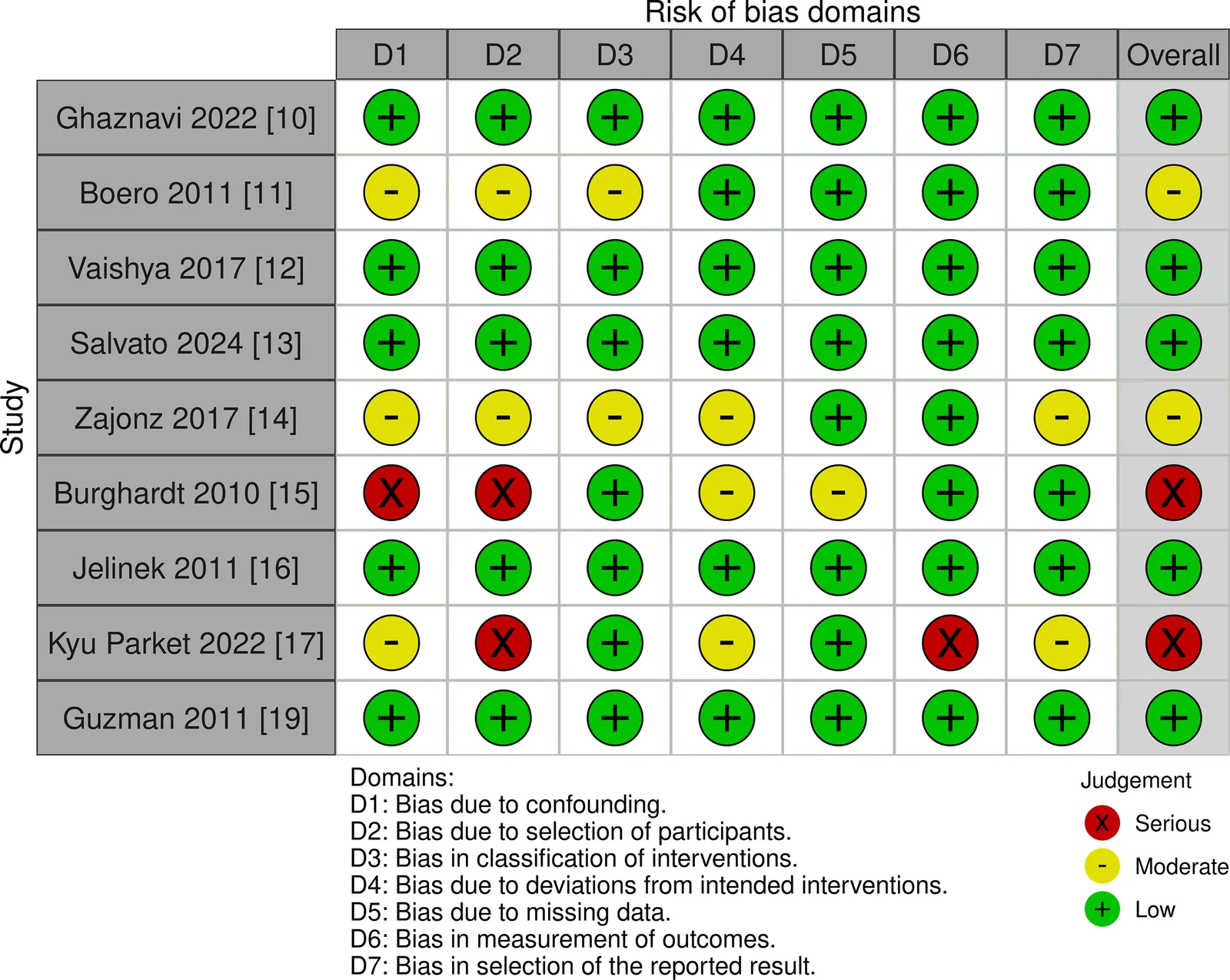
Risk of bias assessments according to ROBINS tools.

## Discussion

The findings of this systematic review confirm that guided growth using tension band plates is an effective and minimally invasive strategy for the correction of idiopathic genu valgum in skeletally immature patients. Radiographic improvements were consistent across studies, with a mean increase of 6.3° in LDFA, a 3.3° decrease in MPTA, and an overall improvement of nearly 7.5° in HKA, demonstrating reliable modulation of the mechanical axis through temporary hemiepiphysiodesis. These outcomes mirror previous reports and reinforce guided growth as a first-line option in growing children [11, 21].

The mean age of 11.7 years in the included cohorts aligns with the window identified in the literature as optimal for maximizing correction and limiting the risk of rebound [22–24]. At this stage of maturation, remaining growth is typically sufficient to achieve correction within 12–18 months, consistent with the mean correction time of 13.2 months observed in our synthesis.

Approximately 60% of procedures were performed at the distal medial femur, reflecting its recognized central role in idiopathic valgus deformity. This predominance is supported by physiological growth patterns, with 65% of longitudinal knee growth originating from the distal femur and 35% from the proximal tibia, which partly explains why femoral hemiepiphysiodesis tends to produce faster and more predictable corrections [25–27]. Nevertheless, literature consistently shows that both LDFA and MPTA may be altered in idiopathic valgus, although the femoral component generally predominates [28]. Of note, femoral plates have been associated with greater postoperative discomfort, likely due to their proximity to soft tissue structures and the quadriceps mechanism [29].

The work of Jae Woo Shim et al. further demonstrates that idiopathic genu valgum frequently results from combined femoral and tibial deformity, with contributions from both segments [28]. This variability, together with the heterogeneity of available evidence, prevents definitive recommendations regarding the optimal site for plate placement. Instead, guided growth should be tailored to the deformity pattern identified on preoperative full-length standing radiographs. When one angular parameter is clearly abnormal while the other remains near normal, the affected segment should be targeted. In more balanced deformities, especially those of moderate magnitude, isolated tibial hemiepiphysiodesis may be advantageous due to better patient tolerance. Combined femoral and tibial hemiepiphysiodesis may be reserved for patients with limited remaining growth or those presenting with more severe deformity, where simultaneous correction may enhance alignment efficiency [28, 30].

Only half of the included studies clearly defined their surgical indications, highlighting ongoing variability in clinical decision-making. The most frequently cited thresholds were a valgus angle >10° on long-standing radiographs or an intermalleolar distance >8 cm [31, 32]. Additional considerations included anterior or lateral knee pain, patellofemoral instability, gait disturbances, and aesthetic concerns. Importantly, recent evidence emphasizes that surgical indication must incorporate not only deformity magnitude but also skeletal maturity and remaining growth potential. Growth prediction models such as those proposed by Paley et al. [33] and Sanders et al. [34] provide reliable estimations without the need for additional radiographic exposure.

A preoperative standing full-length radiograph remains mandatory for accurate deformity analysis and operative planning. Follow-up schedules varied substantially among reports, typically ranging from every 2 to 4 months. Our synthesis and prior evidence [11, 13] suggest that follow-up every 3–4 months provides an appropriate balance between safety and efficiency. Radiographic evaluation at six-month intervals – or earlier if rapid angular changes are suspected – is recommended to guide plate removal and prevent overcorrection. [22, 35] Additionally, at least 12 months of residual growth is required to ensure a meaningful correction before physeal closure [36].

The overall complication rate of 3.6% confirms the favorable safety profile of tension band plates. Overcorrection was the most frequently reported event, followed by superficial infections and isolated cases of recurvatum or screw loosening, all resolving with conservative management. Importantly, no deep infections, physeal arrests, or irreversible deformities were identified, underscoring the minimally invasive and reversible nature of this technique.

This review has several limitations. The inability to perform subgroup analyses based on correction site limits the granularity of the findings. Moreover, the retrospective design of most included studies and the predominance of uncontrolled case series reduce the overall level of evidence and constrain meta-analytic interpretation. Despite these limitations, this review represents the most comprehensive synthesis to date on guided growth using tension band plates for idiopathic genu valgum, providing meaningful insights into radiographic outcomes, surgical indications, and postoperative management.

## Conclusions

Guided growth with tension band plates is a safe, effective, and reversible technique for correcting idiopathic genu valgum in growing children. Surgical indication should rely on both deformity magnitude and remaining growth potential, ensuring that at least one year of growth is available. Accurate identification of the primary deformity site and regular follow-up every 3–4 months enables controlled correction and timely implant removal, minimizing overcorrection.

## Data Availability

This article has no associated data generated.

## References

[1] Gupta P, Gupta V, Patil B, et al. (2020) Angular deformities of lower limb in children: Correction for whom, when and how? J Clin Orthop Trauma 11(2), 196–201. 10.1016/j.jcot.2020.01.008 PMC7026560 32099279

[2] Coppa V, Marinelli M, Procaccini R, et al. (2022) Coronal plane deformity around the knee in the skeletally immature population: A review of principles of evaluation and treatment. World J Orthop 13(5), 427–443. 10.5312/wjo.v13.i5.427 PMC9124997 35633744

[3] Stevens PM (2007) Guided growth for angular correction: a preliminary series using a tension band plate. J Pediatr Orthop 27(3), 253–259. 10.1097/BPO.0b013e31803433a1 17414005

[4] Tirta M, Hjorth MH, Jepsen JF, et al. (2024) Staples, tension-band plates, and percutaneous epiphysiodesis screws used for leg-length discrepancy treatment: a systematic review and proportional meta-analysis. Acta Orthop 95, 415–424. 10.2340/17453674.2024.41104 PMC11257069 39023429

[5] Choi KJ, Lee S, Park MS et al. (2022) Rebound phenomenon and its risk factors after hemiepiphysiodesis using tension band plate in children with coronal angular deformity. BMC Musculoskelet Disord 23, 339. 10.1186/s12891-022-05310-z PMC8994335 35395849

[6] Park H, Park M, Kim SM, et al. (2018) Hemiepiphysiodesis for idiopathic genu valgum: Percutaneous transphyseal screw versus tension-band plate. J Pediatr Orthop 38(6), 325–330. 10.1097/BPO.0000000000000821 27658181

[7] Moher D, Liberati A, Tetzlaff J, et al. (2009) Preferred reporting items for systematic reviews and meta-analyses: The PRISMA statement. Ann Intern Med 151(4), 264–269. 10.7326/0003-4819-151-4-200908180-00135 19622511

[8] DerSimonian R, Laird N (2015) Meta-analysis in clinical trials revisited. Contemp Clin Trials 45(Pt A), 139–145. 10.1016/j.cct.2015.09.002 PMC4639420 26343745

[9] Thorlund K, Imberger G, Johnston BC, et al. (2012) Evolution of heterogeneity (I2) estimates and their 95% confidence intervals in large meta-analyses. PLoS One 7(7), e39471. 10.1371/journal.pone.0039471 PMC3405079 22848355

[10] Ghaznavi A, Baghdadi T, Bagherifard A, et al. (2022) Comparison of eight-plate with reconstruction-plate in temporary hemiepiphysiodesis to correct the idiopathic genu valgum in pediatrics. Arch Bone Jt Surg 10(7), 585–591. 10.22038/ABJS.2022.60663.2990 PMC9382255 36032638

[11] Boero S, Michelis MB, Riganti S (2011) Use of the eight-Plate for angular correction of knee deformities due to idiopathic and pathologic physis: initiating treatment according to etiology. J Child Orthop 5(3), 209–216. 10.1007/s11832-011-0344-4 PMC3100457 22654982

[12] Vaishya R, Shah M, Agarwal AK, et al. (2017) Growth modulation by hemi epiphysiodesis using eight-plate in Genu valgum in Paediatric population. J Clin Orthop Trauma 9(4), 327–333. 10.1016/j.jcot.2017.11.004 PMC6224631 30449980

[13] Salvato D, Chipman DE, Cirrincione P, et al. (2024) Temporary hemi-epiphysiodesis with tension band plates in skeletally immature patients with genu valgum: Faster correction in patients with more than 2 years of expected time of growth remaining. J Child Orthop 18(4), 372–378. 10.1177/18632521241229976 PMC11295371 39100981

[14] Zajonz D, Schumann E, Wojan M, et al. (2017) Treatment of genu valgum in children by means of temporary hemiepiphysiodesis using eight-plates: short-term findings. BMC Musculoskelet Disord 18(1), 456. 10.1186/s12891-017-1823-7 PMC5688618 29141620

[15] Burghardt RD, Herzenberg JE (2010) Temporary hemiepiphysiodesis with the eight-Plate for angular deformities: mid-term results. J Orthop Sci 15(5), 699–704. 10.1007/s00776-010-1514-9 20953936

[16] Jelinek EM, Bittersohl B, Martiny F, et al. (2011) The 8-plate versus physeal stapling for temporary hemiepiphyseodesis correcting genu valgum and genu varum: a retrospective analysis of thirty five patients. Int Orthop 36(3), 599–605. 10.1007/s00264-011-1369-5 PMC3291772 21983939

[17] Park BK, Kim HW, Park H, et al. (2022) Natural behaviours after guided growth for idiopathic genu valgum correction: comparison between percutaneous transphyseal screw and tension-band plate. BMC Musculoskelet Disord 23(1), 1052. 10.1186/s12891-022-05996-1 PMC9719162 36461004

[18] Gottliebsen M, Rahbek O, Hvid I, et al. (2013) Hemiepiphysiodesis: similar treatment time for tension-band plating and for stapling: A randomized clinical trial on guided growth for idiopathic genu valgum. Acta Orthop 84(2), 202–206. 10.3109/17453674.2013.782526 PMC3639343 23485073

[19] Guzman H, Yaszay B, Scott VP, et al. (2011) Early experience with medial femoral tension band plating in idiopathic genu valgum. J Child Orthop. 5(1), 11–17. 10.1007/s11832-010-0310-6 PMC3024485 21415941

[20] Sterne JA, Hernán MA, Reeves BC, et al. (2016) ROBINS-I: a tool for assessing risk of bias in non-randomised studies of interventions. BMJ 355, i4919. 10.1136/bmj.i4919 PMC5062054 27733354

[21] Di Gennaro GL, Trisolino G, Stallone S, et al. (2023) Guided growth technique for epiphysiodesis and hemiepiphysiodesis: safety and performance evaluation. Children (Basel) 11(1), 49. 10.3390/children11010049 PMC10814807 38255362

[22] Zeng JF, Xie YY, Liu C, et al. (2024) Effective time, correction speed and termination time of hemi-epiphysiodesis in children. World J Orthop 15(1), 1–10. 10.5312/wjo.v15.i1.1 PMC10824067 38293262

[23] Ramazanov R, Ozdemir E, Yilmaz G, et al. (2021) Rebound phenomenon after hemiepiphysiodesis: Determination of risk factors after tension band plate removal in coronal plane deformities of lower extremities. J Pediatr Orthop B 30(1) 52–58. 10.1097/BPB.0000000000000786 32732797

[24] Ko KR, Shim JS, Shin TS, et al. (2022) Factors affecting rebound phenomenon after temporary hemiepiphysiodesis and implant removal for idiopathic genu valgum in adolescent patients. J Pediatr Orthop, 42(4), e336–e342. 10.1097/BPO.0000000000002090 35142715

[25] Santos L, Guariento A, Moustoufi-Moab S, et al. (2024) Comparison of tibial and femoral physeal diffusion tensor imaging in adolescents. Pediatr Radiol 54(13), 2243–2253. 10.1007/s00247-024-06073-6 PMC11638376 39516384

[26] Has B, Gurses MS, Altinsoy HB (2023) Evaluation of distal femoral and proximal tibial epiphyseal plate in bone age estimation with 3.0T MRI: a comparison of current methods. Br J Radiol 96(1143), 20220561. 10.1259/bjr.20220561 PMC9975374 36606509

[27] Sepúlveda M, Téllez C, Villablanca V, et al. (2022) Distal femoral fractures in children. EFORT Open Rev 7(4), 264–273. 10.1530/EOR-21-0110 PMC9069856 37931413

[28] Shim JW, Lee SS, Ko KR (2024) Contributions of the distal femur and proximal tibia to idiopathic genu varum and genu valgum in adolescents. Clin Orthop Surg 16(6), 1010–1018. 10.4055/cios24160 PMC11604568 39618518

[29] Fillingham YA, Kroin E, Frank RM, et al. (2014) Post-operative delay in return of function following guided growth tension plating and use of corrective physical therapy. J Child Orthop 8(3), 265–271. 10.1007/s11832-014-0590-3 PMC4142885 24820624

[30] Ballal MS, Bruce CE, Nayagam S (2010) Correcting genu varum and genu valgum in children by guided growth: temporary hemiepiphysiodesis using tension band plates. J Bone Joint Surg Br 92(2), 273–276. 10.1302/0301-620X.92B2.22937 20130322

[31] Masquijo JJ, Artigas C, de Pablos J (2021) Growth modulation with tension-band plates for the correction of paediatric lower limb angular deformity: current concepts and indications for a rational use. EFORT Open Rev 6(8), 658–668. 10.1302/2058-5241.6.200098 PMC8419796 34532073

[32] Kearney SP, Mosca VS (2015) Selective hemiepiphyseodesis for patellar instability with associated genu valgum. J Orthop 12(1), 17–22. 10.1016/j.jor.2015.01.005 PMC4354475 25829756

[33] Paley D, Bhave A, Herzenberg JE, et al. (2000) Multiplier method for predicting limb-length discrepancy. J Bone Joint Surg Am 82(10), 1432–1446. 10.2106/00004623-200010000-00010 11057472

[34] Sanders JO, Khoury JG, Kishan S, et al. (2008) Predicting scoliosis progression from skeletal maturity: a simplified classification during adolescence. J Bone Joint Surg Am 90(3), 540–553. 10.2106/JBJS.G.00004 18310704

[35] Feijoo E, Nihalani S, Sisson H, et al. (2025) Follow-up after hemiepiphysiodesis: Can we do better? J Pediatr Orthop 45(7), e618–e624. 10.1097/BPO.0000000000002962 40162483

[36] Siemensma MF, van Bergen CJA, van Es EM, et al. (2023) Indications and timing of guided growth techniques for pediatric upper extremity deformities: A literature review. Children (Basel) 10(2), 195. 10.3390/children10020195 PMC9954695 36832323

